# Some Observations Concerning Hydro-Chlorate of Cocaine after Thirteen Months’ Careful Experiments

**Published:** 1886-01

**Authors:** R. O. Cotter

**Affiliations:** Macon, Ga., Late Assistant to the Chair of Eye, Ear and Throat Diseases in Atlanta Medical College


### SOME OBSERVATIONS CONCERNING HYDROCHLORATE OF COCAINE AFTER THIRTEEN MONTHS' CAREFUL EXPERIMENTS.

---

BY R. O. COTTER, M. D. MACON, GA.,

LATE ASSISTANT TO THE CHAIR OF EYE, EAR AND THROAT DISEASES IN ATLANTA MEDICAL COLLEGE.

---

I am fully aware that cocaine is becoming a threadbare subject, yet, as I have noticed no similar observations to the following in your journal, and more especially as I am frequently in receipt of letters from practitioners in this section requesting information respecting the use of cocaine in this or that eye, ear, or throat affection, I take the liberty of presenting these remarks, with the observation that I do not believe that it is time yet to pass full judgment upon cocaine. Its record is yet incomplete. We do not yet know that it may not prove to be a harmful agent. I do not think we can say whether it is best to keep it in the form of crystals or in solutions. I find it more convenient to keep it in solution. Some claim that all solutions soon deteriorate. I procured an ounce of a four per cent. solution while in New Orleans last January, and I am positive that it was perfectly good at the end of five months. This preparation was kept in a covered bottle, carefully corked. I do not know what manufacture of cocaine was used in this preparation. I used other preparations of cocaine during the time of using this, prominent among which was that made by W. H. Schieffelin & Co., of New York. It is not for the purpose of making invidious comparisons between the various manufactures of cocaine, but candor and justice compel me to say that I have used five different manufactures of it, and that of Schieffelin's is the only one which has failed me where others have succeeded, and has also decomposed on my hands. As to color, some claim that the solution should be perfectly colorless, and while I think that an almost colorless solution shows less danger of beginning, or already existing decomposition, a slightly amber tint is not objectionable, provided the *odor* be all right. If a decomposed odor should arise, your cocaine will disappoint you. I cannot think of a less inelegant or more correct comparison than to say that the odor of a decomposed solution resembles that of a "rat's nest." Squibbs, Mercks, and Parke, Davis & Co.'s preparations have given me satisfaction. The solution of P., D. & Co.'s, which I used, showed slightly more color than that of Squibbs', yet P., D. & Co.'s was as good as Squibbs' or Mercks'. The newspapers are having a good deal to say about the harmful effects of cocaine, and while such accounts are usually not to be relied upon, I think it not unlikely that the internal use of it for a length of time would very likely result in a habit resembling the opium habit.

Cocaine injected into the circulation produces some nervous or mental impressions, though, so far as I have observed, it has been nothing serious or even very unpleasant. The cases in which I have used it hypodermically were in enucleations of the eyeball. I have used a four per cent. solution of Mercks' cocaine in three cases of enucleation. In each case I injected twenty-five to thirty drops into the tissues around the ball. In one of these cases the patient exhibited no symptoms of shock. The second, a healthy subject like the first, exhibited some shock and considerable thirst. The third, a delicate man from one of the lower counties in South Carolina. He had been having chills. He showed some mental excitement, and in a few minutes had what is commonly called a dumb chill. In these three cases the pain of the operation amounted to very little.

With a freshly prepared six per cent. solution of Squibbs' cocaine, I recently enucleated the eye of an old gentleman in South Georgia, one of the most honored and intelligent members of our profession. His mental faculties were perfect, yet, owing to his extreme age, 78 years, I preferred to not use ether or chloroform. The old gentleman, though worn down with pain from his eye trouble (an old case of dislocated lens and irido-choroiditis), assured me afterwards that the pain of the operation was really slight.

In this case I used not over twenty-five drops of the solution, but I am very positive that a very decided constitutional impression was made. The patient exhibited much the same pleasant exhilarations of spirits that a stimulating dose of alcohol or opium would have produced.

In superficial operations upon the eye, such as pterygium, it is a good thing to apply weak solutions of cocaine to the wound, for four to six hours afterwards, to reduce the determination of blood and prevent inflammation.

It has always been my custom, after almost any operation, not to allow the patient to leave town for at least two or three days. Yet, I have allowed a few cases to go home immediately after the operation. I recently removed a large pterygium from the eye of a gentleman living about forty miles away. As it was necessary for him to take the train immediately for home, I had him use a two per cent. solution of cocaine on the eye, after the operation, as it was more convenient to use on the cars than applying cold cloths. He came back two days afterward to have the stitches removed, and I found that there was no inflammation present. This same plan was adopted in another case in which I removed a very large pterygium from the eye of a little girl, aged nine years. (By the way, I will mention, that after a close observation of eye diseases for over six years, during four of which I was associated with Dr. A. W. Calhoun, in Atlanta, where we frequently saw over a hundred patients daily, I cannot recall having seen a case of pterygium in a child before). Cocaine was used during and after this operation, and although the child afterwards acknowledged that it was fright and not pain which made her cry, it took three persons to hold her, and made the operation both very tedious and risky. As her uncle, a physician, was with her, I allowed her to leave for home, thirty-five miles distant, immediately, and directed a two per cent. solution of cocaine to be applied, as in the former case. The doctor afterwards informed me that she had no trouble, and that the stitches came away in five or six days, as I wished them to do. It is not often that we can use cocaine in operations upon children. Their fear and movements make too much risk of damaging an eye.

I have seen one case of failure, and I am satisfied it was not owing to fault of the cocaine, nor altogether lack of nerve in the patient. It was a case of acute glaucoma of five days' standing. The tension of the ball was very great, and the pain intense. The vision had gone down from $\frac{10}{50}$ on the second day of the attack, when I first saw him, to nothing on the fifth day. He had refused to submit to any operation until the fifth day, when, more to give him relief from the pain than the hope of restoring his sight, I got his consent, and using the same six per cent. solution which I had used the day before in removing the eye of the physician before mentioned. I gave the cocaine plenty of time to act. (I usually find five minutes or less sufficient.) In this case I waited fully fifteen minutes, and repeated the instillations frequently. I wished to make a large iridectomy, and consequently made the corneal cut and flap as in extraction of cataract. It was with great difficulty that I could even do this much, as the patient screamed and nearly jumped out of bed with pain. The slightest attempt to touch the iris showed that it would be useless to proceed further, and so I had to content myself with letting off the aqueous, and reducing the tension, as I did not wish to risk giving him ether or chloroform, as he had unmistakable heart disease. The tension being relieved, the pain soon eased down, and by a week's time the vision was restored to $\frac{10}{50}$.

I am confident that the failure of the cocaine in this case was largely due to the extreme tension of the ball, preventing proper absorption of the cocaine.

My experience is, that while I have not used a stronger than a six per cent. solution, I have never yet been able to get fully the desired or expected anæsthetic effect upon the iris. Dr. C. R. Agnew, of New York, writes me that he gets good results by waiting fifteen minutes, and frequently repeating the instillations. I used some of this same six per cent. upon the following case one week afterward. A boy ten years old was brought to me from Telfair county. Five and a half years ago he had put an ordinary plum seed up his right nostril. It had become lodged and had finally resulted in serious trouble. The boy could not breathe through this nostril. The seed had set up a nasal catarrh with a very offensive odor and swelling and hypertrophy of the nasal mucous membrane. Using a Wilde's toothed ear forceps (which, by the way, is a most valuable instrument in aural and nasal surgery), I applied a small mop of absorbent cotton saturated in cocaine, and although the nostril was in a very inflamed and painful condition, I gradually inserted the cotton all along through the whole extent of the nostril, and the constringing effect of the cocaine reduced the hypertrophy and tenderness. I could not yet see the seed, but by making a rhinoscopic examination of the posterior nares, I located it just below and back of the inferior turbinated bone. I very readily extracted the seed through the anterior nares, and after giving his parents some directions concerning the treatment of the catarrh, they took him home the same afternoon.

Cocaine has always given me perfect satisfaction in operations for stricture of the nasal duct. Of three cases of this operation last week, I demonstrated its value to Dr. J. P. Stevens, of this city, who was present at one of them. This has always been quite a painful operation without the use of cocaine.

Recently I had the tonsils of three patients to remove in one day—all adults. I alternated with each patient. On one tonsil I would apply a four per cent. solution of cocaine, and on the other I would use none. I applied the cocaine freely, and waited ten minutes for it to take effect. Each operation caused great pain, and I could not perceive that the cocaine, applied in this manner, mitigated the pain at all.

I have generally been disappointed with cocaine where I applied it to irritable throats. When I wished to make a thorough laryngoscopic examination, as in laryngeal phthisis, or ulcerations about the vocal cords, I have applied it with a mop and with an atomizer, but it so happened that I was generally disappointed with it.

It is a most useful remedy when we wish to inflate the tympanum in cases of aural catarrh when there is considerable hypertrophy and tenderness in the nasal tract. Although its effect here is temporary, it gives great relief to such patients who would not readily submit to the insertion of the eustachian catheter.

I have lately been using it in an ointment in cases of hypertrophic nasal catarrh with very great satisfaction.

Very few cases of long standing hypertrophy can be cured without an operation, yet I find the following ointment gives very great relief in a number of cases:

| | |
|---|---|
| ℞—Ol. eucalyptol, . . . . . . . . . . . . | m. v—x. |
| Ext. pinus canadensis (fl.) . . . . | m. viij—xii. |
| Acid carbol. . . . . . . . . . . . . . . | gtt. v—x. |
| Cocaine, four per cent. sol. . . . . . . | ʒ ss.—ʒ iss. |
| (or cocaine crystals, gr. ii—v.) | |
| Oil of rose, . . . . . . . . . . . . . . | gtt. ij. |
| Vaseline, . . . . . . . . . . . . . . . | ℥ ss.—℥ i. |
| M. F. ungt. | |

(I sometimes add 1 ʒ iodoform, finely pulverized, if there is much fetor.)

I regard it as one of the best adjuncts I have used in the treatment of this affection, especially if the hypertrophy is too far back to be easily gotten at to operate upon.

This ointment should be applied twice daily thoroughly in the nostrils with a camel's hair brush, or a strand of absorbent cotton as large as the finger, and four or six inches long, saturated with the ointment, and pack thoroughly in one nostril at night, allowing it to remain until morning, the other nostril to be treated in the same manner the following night.

I have a number of patients now using this ointment, and they all express themselves as greatly pleased with its action. One of them, a highly intelligent physician, who has a post nasal catarrh with posterior hypertrophy, introduces the ointment behind the posterior nares with his fingers. He is confident that he has found nothing which has afforded him so much relief.

I am aware that harm might possibly come from the long-continued use of cocaine in this way, and I am, as yet, very guarded in its use in this way, though so far I have not seen any harmful results.

In cases of inflammation of the drum membrane, where suppuration is imminent, I have found that frequent applications of a solution of cocaine, along with other treatment, has greatly mitigated the pain, and, I think, checked the inflammation.

I consider cocaine the most valuable adjunct in ear, throat, nasal and eye surgery that has yet been discovered.

